# Short‐term reservoir draining to streambed for juvenile salmon passage and non‐native fish removal

**DOI:** 10.1002/eco.2096

**Published:** 2019-07-03

**Authors:** Christina A. Murphy, Gregory Taylor, Todd Pierce, Ivan Arismendi, Sherri L. Johnson

**Affiliations:** ^1^ Department of Fisheries and Wildlife Oregon State University Corvallis OR; ^2^ Willamette Valley Project US Army Corps of Engineers Lowell OR; ^3^ Pacific Northwest Research Station US Forest Service Corvallis OR

**Keywords:** drawdown, invasive species, operational measure, outmigration, reservoir management, river, smolt, stream connectivity

## Abstract

Fish passage out of reservoirs is a critical issue for downstream movement of juvenile salmonids and other migratory species. Reservoirs can delay downstream migrations by juvenile salmon for months or years. Here, we examine whether a novel management activity implementing annual short‐term draining of a reservoir to streambed improves timely downstream migration of juvenile salmonids. We analyse 12 years of fish capture data from a screw trap located downstream of Fall Creek Reservoir (Oregon, USA) to examine changes in timing of passage out of the reservoir and to compare fish species composition pre‐ and post‐draining. We observed a contraction in the timing of downstream migration for juvenile Chinook Salmon and reduction of yearlings in years following draining. We suggest that briefly draining the reservoir to streambed leads to reduced abundance of warm‐water invasive fishes in the reservoir after it refills. These changes could decrease predation and shift competition between invasive and resident riverine‐adapted native fishes in the reservoir. Collectively, our findings suggest that this low‐cost reservoir management option may improve passage and connectivity for juvenile Chinook Salmon while also decreasing the abundance of invasive fish species in the reservoir. This case study underscores the crucial need for further evaluations of reservoir draining in other systems and contexts.

## 1 INTRODUCTION

Large dams (>15‐m height) have long been recognized as having profound implications for fish migration. Large dams serve as barriers to both up‐ and down‐stream migration, with severe consequences for migratory species (Stanford & Ward, 2001; Stone, 2011). In the Pacific Northwest of North America (PNW), large dams and reservoirs have often been constructed on anadromous salmon‐bearing rivers. As a consequence of impediment by large dams, these anadromous species have lost access to historical habitats and many of them are now listed under the U.S. Endangered Species Act (NOAA Fisheries, 2008). Even when adults are able to access upstream reaches by fish ladders or trap and haul operations, large dams and reservoirs often result in high mortality and delayed downstream migration of juvenile salmon both because of difficulty locating downstream outlets and because outlets that attract fish may cause injury (Korn & Smith, 1971; Raymond, 1979; Schilt, 2007). Modification of dam outlet structures or construction of new structures to allow more effective downstream passage of fish are expensive (Williams, Armstrong, Katopodis, Larinier, & Travade, 2012) and can lead to additional problems with invasion of undesirable non‐native species (Fausch, Rieman, Dunham, Young, & Peterson, 2009).

The establishment of invasive species in these areas is often facilitated by the creation of the artificial lentic, reservoir habitats associated with large dams (Havel, Lee, & Zanden, 2005; Vander Zanden, Lapointe, & Marchetti, 2015). In the PNW, invasive fish species in reservoirs include top piscivores such as Largemouth Bass (*Micropterus salmoides*) and crappie (*Pomoxis* sp.) that co‐occur with native juvenile salmonids (Keefer et al., 2013). The abundance of invasive piscivores associated with large dams and reservoirs in this region also raises concerns about juvenile salmonid survival due to potential increases in predation (Monzyk, Romer, Emig, & Friesen, 2014). This predation risk likely acts in concert with passage problems negatively impacting juvenile salmon.

Because of the low success and high cost of construction of downstream passage facilities, especially in highly fluctuating reservoirs, temporary draining of reservoirs to streambed during outmigration periods has been proposed. This is considered an operational alternative to improve downstream passage for juvenile Chinook Salmon (*Oncorhynchus tshawytscha)* at some PNW dams (Tiffan, Garland, & Rondorf, 2006; United States Army Corps of Engineers [USACE], 2015). By fully opening a dam outlet (e.g., gate valve), removing a physical barrier and temporarily converting the lentic reservoir into a lotic river, draining to streambed could improve the ability of juvenile salmon to locate the reservoir outlet and to pass downstream with minimal obstruction, high survival and without handling. This is effectively reservoir flushing, and may be accompanied by a washout of sediments (Schenk & Bragg, 2014) as well as other fish species. Although there is some history of draining for passage and dam maintenance (Anderson, 2007), this strategy has not previously been evaluated for short‐ and longer‐term impacts to the reservoir community.

Here, we quantify changes in timing of downstream passage of fish and in the fish community structure before and after brief fall annual draining of the reservoir to streambed using 12 years of data from fish collections downstream of a PNW reservoir. We also examine whether draining to streambed was followed by changes in the number of returning adult Chinook Salmon. We hypothesize that short‐duration reservoir draining to streambed can be used to 1) increase the effectiveness of downstream passage of juvenile salmon, improving numbers of individuals available to recruit to the adult population. We predict that more effective passage will be evidenced by a reduction in passage in the months after reservoir draining to streambed (juvenile salmon will have already moved downstream) and that adult numbers will increase in subsequent years. 2) We also hypothesize that reservoir draining to streambed will ultimately reduce abundances of warm‐water invasive species in the reservoir. We expect warm‐water species to be highly sensitive to the temporary lotic conditions experienced during draining, which might favour a transition to a community dominated by cool‐water riverine‐adapted native species in the reservoir over time. We expect a reduction in abundance of warm‐water invasive species will result in shifting composition from invasive–dominated catches to native–dominated catches in the downstream screw trap. This work is an important first step in evaluating a novel management activity that could serve as an alternative to modification of dam outlet structures and may offer a cost‐effective solution to downstream passage and invasive species management.

## 2 METHODS

### 2.1 Study site

**Figure 1:**
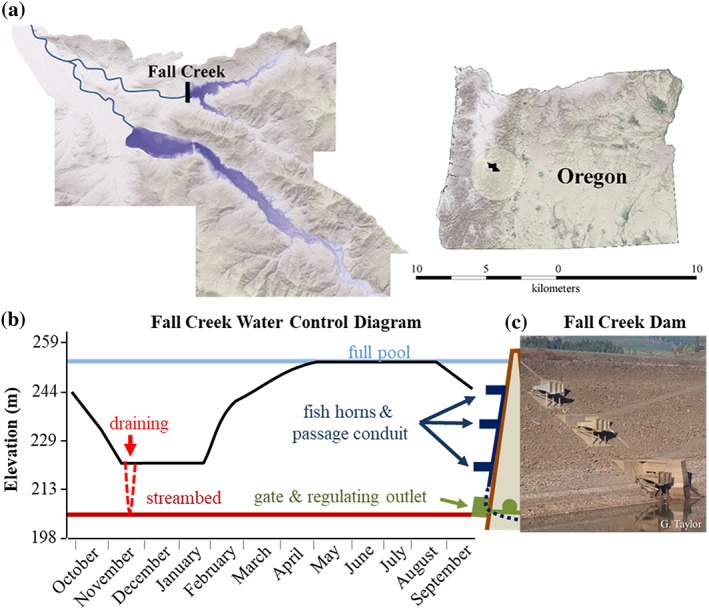
Physical characteristics of Fall Creek Dam and Reservoir. (a) Map of dam (black line) and reservoir (blue) location in the upper Willamette River Basin, Oregon, USA. (b) “Water Control Diagram” showing water levels in the reservoir over a water year (Oct to Sept) with idealized draining to streambed (dashed red line), streambed (solid red line) and sidecut dam overlay depicting outlets (fish horns, dark blue; gate and RO structures, green). (c) Photo of reservoir side of Fall Creek Dam showing water outlets and fish passage structures

This study was conducted in Fall Creek Reservoir, which was constructed in 1966 on a tributary of the Willamette River, western Oregon (dam height = 55 m, water surface elevation range = 205–254 MAMSL, reservoir area = 736 ha, reservoir volume = 0.142 km^3^; Figure 1, USACE, 2017). This and other large dams in the Willamette River Basin are operated by the USACE for multiple objectives that include reducing flood risk, improving downstream water quality, providing onsite recreation, and supplying downstream water for irrigation, navigation, and fish and wildlife management (USACE, 2014). Balancing these objectives typically results in a low water elevation in the winter for flood control, refilling in the spring, maximum water elevation for summer recreation, and a drawing back down to low water elevation in the fall (Figure 1). There are no additional complete barriers to salmon migration between Fall Creek Dam and the Pacific Ocean. However, salmon must pass Willamette Falls on the mainstem Willamette River (178 river miles below Fall Creek dam); a basalt waterfall where a canal and locks were constructed in the late 1800s, along with a fish ladder that was replaced in 1971 (ODFW, 2019).

The fish species composition of Fall Creek Reservoir, which includes ESA‐listed juvenile Chinook Salmon, has historically been dominated by invasive Bluegill, (*Lepomis macrochirus*), Black Crappie (*Pomoxis nigromaculatus*), and Largemouth Bass (*Micropterus salmoides*; Keefer et al., 2013). A complete list of observed fish taxa is provided in Table 1. Hatchery juvenile spring Chinook Salmon were stocked into Fall Creek Reservoir until 2001 (Jeff Ziller, personal communication); therefore, reservoir outmigrating juvenile Chinook Salmon included in this study originated from natural production by wild or hatchery adults. Upstream fish passage for returning adult salmon at Fall Creek Dam consists of trap and haul operations; adult salmon are captured at the base of the dam, loaded, transported, and released upstream of the reservoir using tank trucks. Although hatchery‐originated smolts were released into Fall Creek downstream of Fall Creek Dam through 2007, beginning in 2009 only wild returning adult salmon were transported upstream of the dam and released for spawning in the headwaters of Fall Creek.

**Table 1:** Fish species from Fall Creek Reservoir, captured in a screw trap deployed below Fall Creek Dam from 2006 to 2017. Taxa include native (top, white background) and invasive (bottom, grey background) species. Groups indicate genera that contain multiple species and/or taxa that were not consistently distinguished and were pooled for analyses (e.g., bullheads)

Designation	Common name	Scientific name	Group
Native	Chinook Salmon	*Oncorhynchus tshawytscha*	
	Largescale Sucker	*Catostomus macrocheilus*	
	Sculpin	*Cottus* sp.	sculpin
	Lamprey	*Lampetra* sp.	lamprey
	Cutthroat Trout	*Oncorhynchus clarkii*	
	Rainbow Trout	*Oncorhynchus mykiss*	
	Mountain Whitefish	*Prosopium williamsoni*	
	Northern Pikeminnow	*Ptychocheilus oregonensis*	
	Longnose Dace	*Rhinichthys cataractae*	
	Speckled Dace	*Rhinichthys osculus*	
	Redside Shiner	*Richardsonius balteatus*	
Invasive	Yellow Bullhead	*Ameiurus natalis*	bullhead
	Brown/Black Bullhead	*Ameiurus nebulosus/melas*	bullhead
	Bluegill	*Lepomis macrochirus*	
	Largemouth Bass	*Micropterus salmoides*	
	White Crappie	*Pomoxis annularis*	crappie
	Black Crappie	*Pomoxis nigromaculatus*	crappie

To facilitate downstream passage of salmonids, Fall Creek Dam was constructed with “fish horns.” These are nine horn‐shaped outlets arranged in groups of three at three elevations on the upstream face of the dam (Figure 1). To migrate downstream, fish must either locate or move through these fish horns or through the dam regulating outlet at the base of the dam; spillway structures are reserved only for emergencies at this location. Korn and Smith (1971) noted that the fish horns at Fall Creek Reservoir were not used by the majority of juvenile Chinook Salmon to migrate downstream. A recent study (Normandeau Associates, Inc., & Pierce, 2014) re‐evaluated the performance of the fish horns and suggested they may have high immediate (56%) and delayed (89%) mortality rates for juvenile subyearling Chinook Salmon outmigrating in the fall. Juvenile Chinook Salmon grow larger in Fall Creek Reservoir than in nearby streams (Monzyk et al., 2014), and their large size may contribute to increased injuries and associated mortality.

### 2.2 Reservoir draining to streambed

To remedy problems with limited passage through the fish horns (Korn & Smith, 1971), brief draining of Fall Creek Reservoir to streambed occurred annually from 1969 until 1979, and once more in 1989, after which reservoir operations changed to maintaining winter reservoir elevations at the “minimum conservation pool” (15‐m deep; Figure 1). Contemporary reservoir draining to streambed was initiated in November 2011 and has occurred annually since. The duration of each annual contemporary draining to streambed event is approximately 1 week but varies depending on inflow conditions (Figure S1). Prior to the contemporary streambed draining, the composition of fishes in Fall Creek Reservoir roughly resembled those found in other nearby reservoirs (Table 1).

### 2.3 Fish trapping downstream of the dam

**Figure 2:**
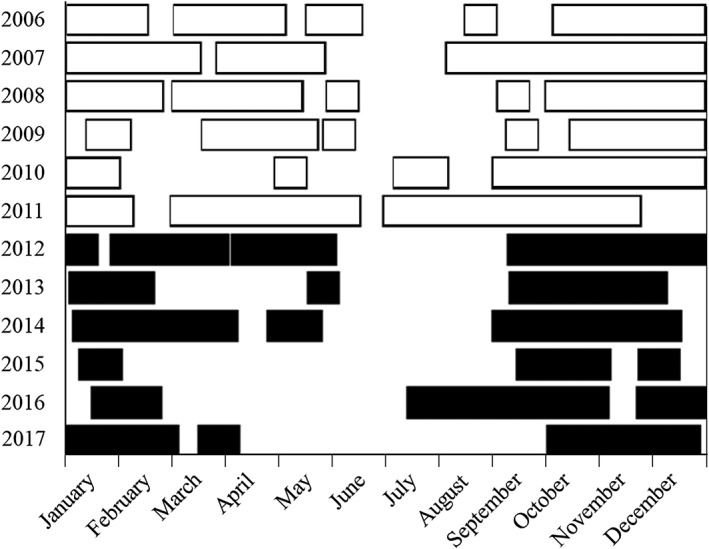
Timing of screw trapping operations when fish were being captured below Fall Creek Dam in the years before (white) and after (black) the initiation of draining to streambed

From 2006 to 2017, USACE trapped fish 165 m downstream of Fall Creek Dam using a 2.44‐m diameter rotary screw trap (EG Solutions, Corvallis, OR). The trap was generally deployed year‐round, although low discharge in summers frequently precluded operation between June and August (Figure 2). Trapping was also not possible for short periods during reservoir draining when the reservoir water level reached streambed, due to clogging from high sediment loads. Efficiency tests for trapping of fish noted up to 12.5% trap efficiency with higher efficiencies generally observed for live fish and during higher water discharge periods (G. Taylor, unpublished data). USACE personnel emptied the trap daily or every other day during operations in accordance with Oregon Department of Fisheries and Wildlife permits 15228, 16345, 17137, 17862, 18646, 19466, 20086, and 21274. Body sizes of juvenile Chinook Salmon collected were measured (fork length; mm) before fish were released to continue downstream migration. Body sizes of other fish species were collected as time and personnel permitted.

### 2.4 Returning adult Chinook Salmon

When adult Chinook Salmon return to Fall Creek Dam, they are trapped, counted, transported via truck upstream of the reservoir, and released into Fall Creek. Adults return to Fall Creek from April through October and spawn in the late summer and early fall. Fry typically emerge from redds the following winter and spring. The adults that were transported upstream in 2010 produced the subyearlings that experienced the first contemporary reservoir draining to streambed event in 2011. This cohort would return to Fall Creek as 3‐ to 6‐year‐old adults between 2014 and 2017, with the majority expected to return as 4‐year olds in 2015 (Claiborne, Fisher, Hayes, & Emmett, 2011; Johnson & Friesen, 2013). Because the number of adults returning are influenced by numerous factors, including estuarine and ocean conditions, we compared the number of adult Chinook Salmon returning to Fall Creek Dam with the number of adults passing Willamette Falls fish ladder on the mainstream Willamette River (RM 27; 178 river miles below Fall Creek Dam. This is the only barrier downstream of Fall Creek Dam, and passage through the ladder is volitional. Comparing the number of adults returning to Fall Creek with numbers of those returning to the larger basin, above Willamette Falls, allows us to adjust for interannual variation attributable to conditions beyond the management of Fall Creek Dam.

### 2.5 Statistical methods

Data were cleaned to standardize coding across years and analysed using *R* Statistical Computing software (R. Core Team, 2014) and the lubridate (Grolemund & Wickham, 2011) package to facilitate time span comparisons. Inconsistent gaps in trap operations precluded the use of time series forecasting. Instead, because of potential differences in trap operation and efficiencies over time that would make a duration based effort correction inaccurate, our analyses focus on relative proportions from daily and monthly captures and on changes in annual cumulative distributions (empirical cumulative distribution function, *R*), metrics that should be more robust to variability and data gaps than fish counts. For evaluation of timing of juvenile Chinook Salmon passage, we used data that included complete subyearling cohorts (i.e., April, when fry first start to enter the reservoir, to March, 2006–2017). Monthly and annual proportions and species–specific sizes were compared for years before and after reservoir draining to streambed using unpaired Wilcoxon tests implemented in *R* with the ggpubr package (Kassambara, 2018). We used the same methods to evaluate changes in the abundance and size structure of invasive species. As reservoir management outside of draining has not changed, we assume that changes in screw trap data reflect changes of the fish community inside of the reservoir. In addition to the proportion of invasive species with respect to native species, which we expect to be less affected, we compared before and after abundance and size within each species as described above. Numeric counts were highly variable across years but are presented in the case of species that were no longer detected in years following draining events.

## 3 RESULTS

### 3.1 Juvenile Chinook Salmon downstream passage

**Figure 3:**
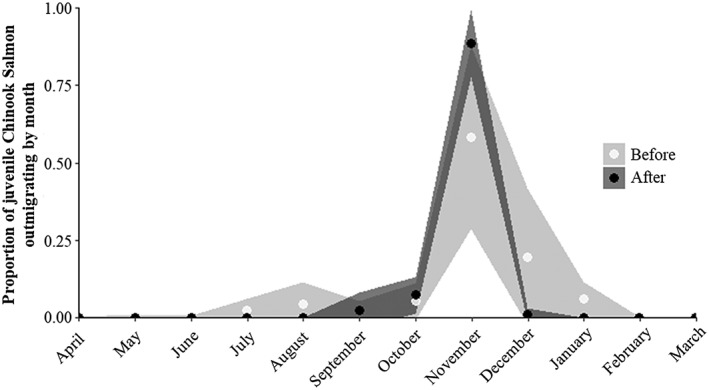
Mean proportion of juvenile Chinook Salmon larger than 60‐mm FL captured in the outmigrant screw trap between April and March (which corresponding to timing of juvenile Chinook Salmon cohorts). Shaded area represents ±1 SD. Open symbols represent the years preceding reservoir draining to streambed (2006–2011) whereas black symbols represent years that included draining to streambed (2011–2017)

Before draining, 95% of the juvenile Chinook Salmon had migrated downstream through the dam by January 4 with some juveniles observed downstream in February. In the years with draining to streambed, 95% of juvenile Chinook Salmon had passed through the dam by November 18, a significant contraction in the later end of the distribution (unpaired two‐tail *t* test, *p* < .01; Figure 3, Table 2). The median passage date for juvenile Chinook Salmon before and after draining has not changed nor has the difference between the median and 75th percentile. However, the variability of the timing of the 75th percentile was reduced after reservoir draining to streambed. Though few to no fish passed after draining treatments in November, the timing of passage was variable before draining. We found no significant differences in the days elapsed between the 5th or 25th percentiles of the cumulative distribution compared to the median. Changes in timing were not accompanied by significant changes in the maximum Chinook Salmon size observed annually moving downstream nor in counts per year, though there was a trend towards smaller salmon, consistent with a reduction of reservoir‐reared yearlings (Figures S2 and S3).

**Table 2:** Mean ± SD of percentile timing (5%, 25%, 50%, 75%, and 95%) for juvenile Chinook Salmon passing downstream (April to March)

Percentile	Date before streambed draining (2006–2011)	Date after streambed draining (2011–2017)	Days from the median passage date before draining	Days from the median passage date after draining	*p* value (*t* test) for change in days from median
5th	October 14th (±40 days)	October 20th (±16 days)	40 (±26)	36 (±10)	0.41
25th	November 8th (±21 days)	November 13th (±9 days)	15 (±2)	13 (±2)	0.06
50th	November 23rd (±12 days)	November 15th (±10 days)	‐	‐	‐
75th	December 4th (±18 days)	November 17th (±10 days)	11 (±13)	2 (±1)	0.14
95th	January 4th (±21 days)	November 18th (±10 days)	42 (±20)	3 (±1)	<0.01

*Note*. Two‐tailed *t* test compared the number of days from the median before (April 2006–March 2011) and after (April 2011–March 2017) the reservoir draining to streambed

### 3.2 Invasive fish species downstream passage

**Figure 4:**
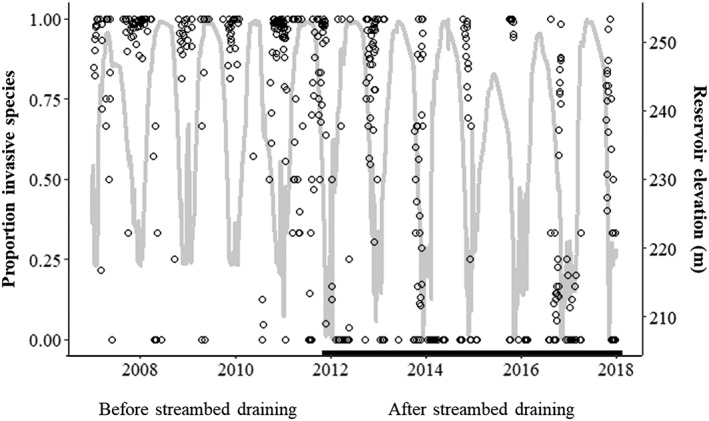
Invasive species as a proportion of the total daily catch (open circles) before and after draining to streambed. The thicker line along the X‐axis represents the period including reservoir draining to streambed. Water surface elevation in the reservoir is represented by the light grey line and is plotted against the secondary Y‐axis

**Figure 5:**
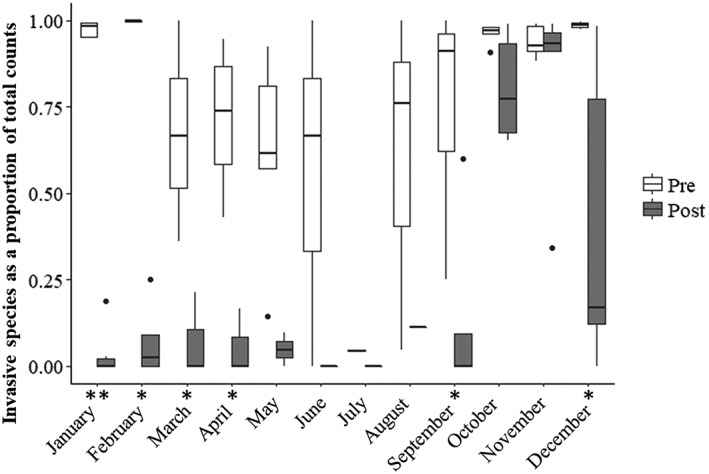
Proportion of invasive fish species (number of invasives per total catch) by month in years before (light) and after (dark) draining to streambed. Boxes represent median and 25th to 75th percentiles, whiskers represent the largest and smallest values within 1.5 * the interquartile range (distance from the 25th to 75th percentile). Stars indicate significant differences before and after contemporary reservoir draining to streambed events (unpaired Wilcoxon Test, *R*, ^*^< .01, ^**^< .001)

**Figure 6:**
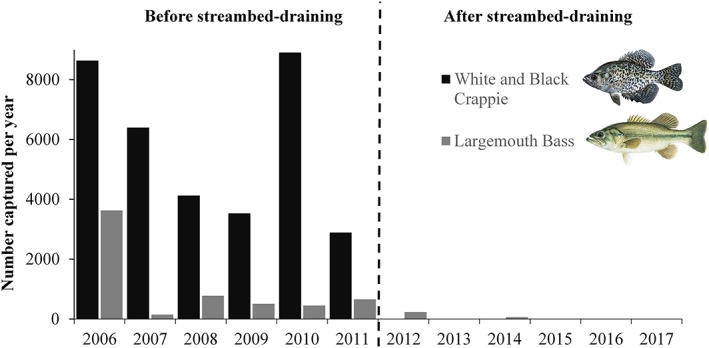
Total number of invasive piscivores captured in the screw trap below Fall Creek Reservoir by year before and after the initiation of reservoir draining to streambed events (dashed line). Fish images © Joseph R. Tomelleri, used with permission

The proportion of invasive species of the total number of fish exported daily across years decreased over time after 2011 following the initiation of reservoir draining to streambed (Figure 4). In addition, the proportion of invasive species exported by month decreased significantly in September and for months between December and April (unpaired Wilcoxon tests, *p* < .01). Trends in downstream passage shifted towards the cold season (e.g., October, November, and December; Figure 5). The capture of invasive piscivores showed the greatest change with continued annual draining to streambed events; captures of crappie, Largemouth Bass, and bullhead catfish comparing years before the 2011 draining event to the years after (Figure S3). Largemouth Bass and White and Black Crappie were no longer captured in the screw trap after 2014 (Figure 6).

Shifts in sizes of fishes occurred in the years following draining to streambed (Figure S3). Smaller maximum sizes of Bluegill and crappie were noted in years with draining events (unpaired Wilcoxon test by species, *p* < .01). We also observed nonsignificant trends towards smaller Largemouth Bass and bullhead. Conversely, some native species including Cutthroat Trout (*Oncorhynchus clarkii*), Rainbow Trout (*Oncorhynchus mykiss*), and Longnose Dace (*Rhinichthys cataractae*) showed trends of larger maximum sizes over this same period (Figure S3).

### 3.3 Adult spring Chinook Salmon returns

**Figure 7:**
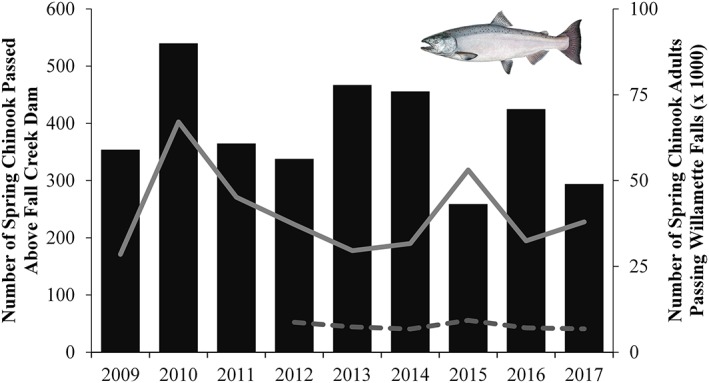
Naturally produced adult spring Chinook Salmon passed above Fall Creek Dam (bars). Lines represent the total (solid) and wild (dashed, beginning in 2012) spring Chinook Salmon passing Willamette Falls on the mainstream Willamette River, 288 km downstream of Fall Creek Dam. Assuming most adults are roughly split across age 4 and 5 (Johnson & Friesen, 2013), the first reservoir draining to streambed cohort (2011) should have returned in 2015 and 2016. Fish image © Joseph R. Tomelleri, used with permission

To address whether the reservoir draining had longer term effects on Chinook Salmon populations, we examined the number of adults returning to Fall Creek Dam starting in 2015. These adults would include the first cohort of juveniles exposed to draining. From 2015 to 2017, 259–425 adults returned per year. Numbers were comparable to prior years, when the median number of returning adults was 410 (range of 338–540). Returns were low in 2015; it was a drought year (Figure 7). To qualify the year–to–year variability, the proportions of adult Chinook Salmon returning to Fall Creek were compared with returns throughout the Willamette Basin, as measured at Willamette Falls. Similar proportions of Fall Creek adults were observed from 2015 to present (0.6–1.7% of total Willamette Falls counts) compared with returning adults pre–draining (2009–2014; 0.9–2.0%).

## 4 DISCUSSION

This study shows that short‐term draining of a reservoir during late fall increases downstream passage efficiency for juvenile Chinook Salmon. Draining also passes non‐native competitors and predatory fish out of the reservoir, which likely reduces their densities in the reservoir and their impact on incoming Chinook Salmon fry the following spring. However, the increased outmigration has not yet resulted in increased returns of Chinook Salmon adults for spawning upstream of the dam and reservoir.

The absence of late migrants suggests that a high proportion of juvenile Chinook Salmon move through the dam during reservoir draining to streambed, leaving few to no juveniles remaining in the reservoir for outmigration later in winter or as yearlings. Because the juvenile Chinook Salmon grow quickly in reservoirs, and the fish horns in the dam result in injuries, outmigration as juveniles through the regulating outlet, rather than the horns, appears to lead to reduced mortality for young Chinook Salmon (Nesbit et al., 2014). These factors support draining to streambed as an “effective passage” option, defined by a high proportion of outmigrating juvenile Chinook Salmon passing through the dam with low passage‐related mortality.

Draining to streambed may also help to reduce reservoir‐related predation risk for juvenile Chinook Salmon through multiple mechanisms. In addition to changes in the abundance of invasive fishes being exported from the reservoir with draining, recent evidence indicates that both native and invasive reservoir resident piscivores switch diets to use lower trophic level sources, consistent with reduced prey fish densities and optimal foraging theory, following draining to streambed events (Murphy, Arismendi, Taylor, & Johnson, 2019). Thus, each spring following draining, juvenile Chinook Salmon may be entering a reservoir with a reduced predator density, and those remaining predators may be focused on capturing lower trophic level food items.

While the downstream export of large numbers of invasive fishes during draining is of concern for downstream river food webs, the same species have been passing through the dam throughout the year each year. The shift in timing of export of invasive species might lower their survival downstream, especially in river networks where conditions during the periods of export could be less favourable to their establishment (e.g., export of warm‐water lentic fish downstream during the winter months with high discharge and cold conditions).

Although reservoir draining appears to reduce mortality for juvenile Chinook as they pass through the dam, as well as provide ecological benefits, such as a reduction in the size and number of invasive predatory fish, it is unclear what the long‐term implications of this pulsed downstream migration will be for maintaining a diversity of Chinook Salmon life histories (see Schroeder, Whitman, Cannon, & Olmsted, 2015). After filling of Fall Creek Reservoir, as observed for other reservoirs (Romer, Monzyk, Emig, & Friesen, 2013), the majority of juvenile spring Chinook Salmon have exhibited downstream passage through the dam in the fall after spring and summer rearing; a smaller proportion of juvenile salmon outmigrated throughout the year as fry, subyearlings, or yearling fish. In years without draining, juveniles continued to exit the dam past the fall months, even when water levels increased above the typical winter minimum pool. Draining results in the absence of these late migrants, including subyearlings passing in winter and yearling fish. Trade‐offs will need to be weighed, given that Chinook Salmon life histories have likely already been modified by the dams and given potential benefits of the reservoir draining on reduced juvenile mortality.

We expected to see an increase in numbers of returning Chinook Salmon adults as the cohorts that were passed downstream by reservoir draining have matured and returned to spawn. Unexpectedly, the numbers of returning adults have stayed relatively constant. One explanation could be that there may not yet have been enough time to see responses, including a reduction of invasive predators in the reservoirs. Declines in invasive fish appear lagged for a year or two following initial draining to streambed and the potential increased survival of juvenile Chinook Salmon from reduced predators in the reservoir would also be delayed. A second explanation could be that the increased number of juvenile (fry to subyearling) outmigrant Chinook Salmon may not be enough to affect adult survivorship. The ratios of adults returning to Fall Creek did not increase relative to returns to the Willamette River basin, as measured at Willamette Falls, suggesting that ocean conditions may not fully explain the lack of response. Ocean conditions experienced by juveniles are known to be the main drivers of adult Chinook Salmon returns (Dale, Daly, & Brodeur, 2017). However, we expect that outmigrant Chinook Salmon juveniles produced in other tributaries above Willamette Falls to experience similar or shared ocean conditions. Adult Chinook Salmon in this basin have also been documented to have highly variable annual prespawn mortality rates (Bowerman, Roumasset, Keefer, Sharpe, & Caudill, 2018). These sources of interannual variability could cause complex relationships between juvenile production, passage survival, and adult returns. Even if higher adult returns do not follow from this shift in management, high survival and effective passage out of reservoirs are critical components needed to ensure anadromous runs of Chinook Salmon are able to be sustainable.

Cultural resource protection, reservoir‐based recreation, and safety are critical considerations for reservoir management practices. Additional challenges specific to the implementation of draining could be similar to dam decommissioning and would include short term changes to downstream water quality, export of sediment, and increases in downstream turbidity. Potential longer‐term consequences would occur for off‐channel habitats if sediments are deposited in the margins and not transported downstream by later high discharge conditions (Kondolf et al., 2014). Sediments from a very productive reservoir could have the potential to deoxygenate downstream reaches. However, our observations of draining of Fall Creek Reservoir, an oligo‐mesotrophic system, suggest that the downstream water quality impacts may be short‐lived. Regardless, reservoir trophic status and amounts of sediment to be mobilized should be considered before draining. Timing of draining is also important to consider. In strongly seasonal environments, such as the PNW, a brief winter draining compared with a brief spring or summer draining would be expected to have very different consequences both within the reservoir and downstream. Concerns over the export of invasive species may be greater in some regions. In the PNW, invasive species are most associated with lentic habitats and are regularly exported from reservoirs regardless of draining (Keefer et al., 2013). Even so, they appear to be outcompeted in the upper Willamette River downstream of the dam described here (Williams, 2014) and the observed shift in export timing to unfavourable seasons may reduce risk. Finally, the large water level fluctuations associated with draining could be of concern for aquatic communities in reservoirs typically operated to have stable water levels, as even comparatively small water level fluctuations can have large consequences for lentic systems (Wantzen et al., 2008).

New options for reservoir management are increasingly under consideration as managers work to reduce ecological impacts of the dam and reservoirs on native species (Chen & Olden, 2017; Poff & Schmidt, 2016). Water levels in large reservoirs are often lowered for reasons other than downstream passage (e.g., for repairs or during drought years). These can provide unique opportunities that could be extended to additional studies of draining to streambed, especially to examine the impacts on reservoir conditions and resident fishes. Considering that draining may provide a low‐cost option to improve passage and connectivity while also exporting invasive species, we highlight the critical need for further evaluations in other systems and contexts. Draining a reservoir to streambed is an interesting management alternative to aid downstream passage of juvenile salmon through large dams, and it needs to be evaluated on a case‐by‐case basis before broad implementation. For example, considering the variability in types of dam outlet structures and potential loss of hydropower generation during the draining and refill periods. Finding more ecologically sound reservoir management practices is a growing concern as we balance the societal benefits of large dams with their ecological costs and draining offers one option in a broad management portfolio.

## Supporting information

Figure S1. Water surface elevations in Fall Creek Reservoir through time highlighting timing and duration of draining to streambed.
Figure S2. Annual maximum observed fish sizes by species before and after initiation of draining to streambed.
Figure S3. Annual counts of juvenile Chinook Salmon and other species before and after initiation of draining to streambed.
