# Supplementary material for: Short‐term reservoir draining to streambed for juvenile salmon passage and non‐native fish removal
Source: Ecohydrology. 2019 Jul 3;12(6):e2096. doi: 10.1002/eco.2096 (PMC6853229; doi:10.1002/eco.2096)
Supplement: Supplementary file 1 — Figure S1. Water surface elevations in Fall Creek Reservoir through time highlighting timing and duration of draining to streambed. Figure S2. Annual maximum observed fish sizes by species before and after initiation of draining to streambed. Figure S3. Annual counts of juvenile Chinook Salmon and other species before and after initiation of draining to streambed. [file ECO-12-na-s001.pdf]

Supplementary material:

A)

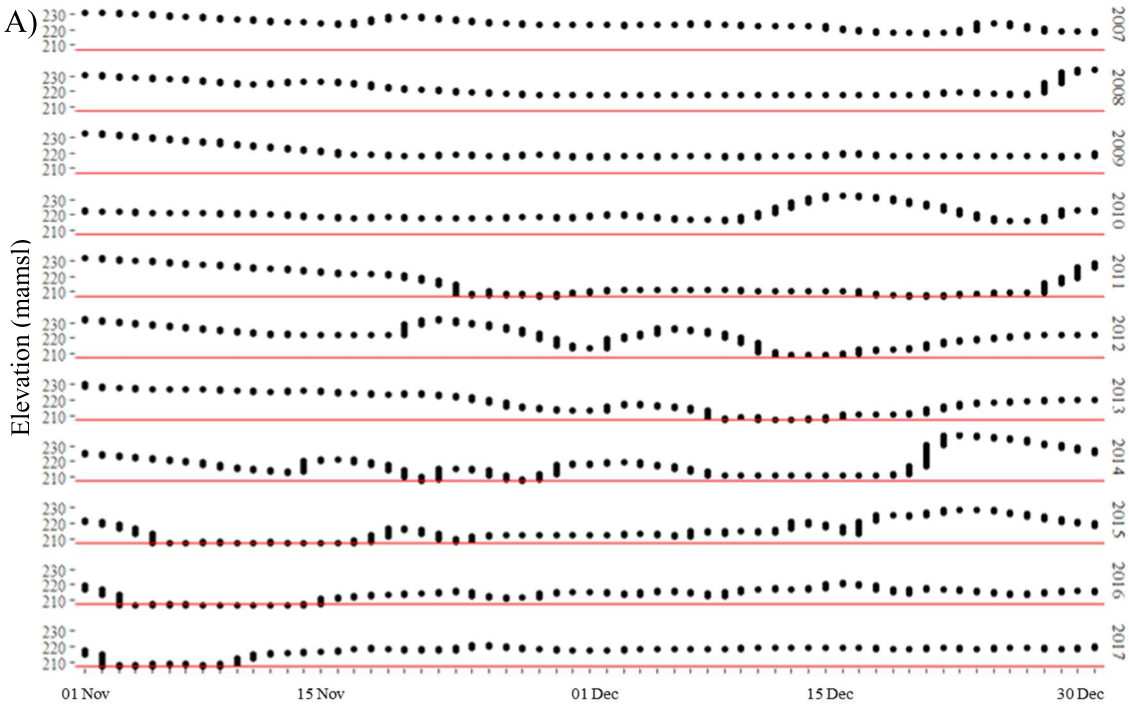

B)

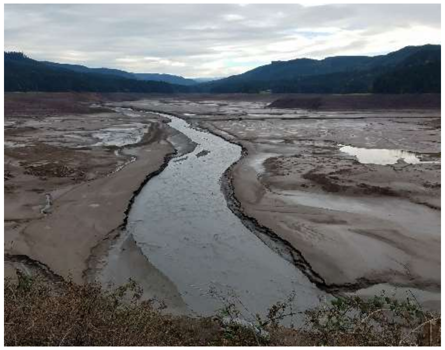

C)

Year	Days below 207.6 mamsl
2011	7
2012	0*
2013	6
2014	1
2015	7
2016	12
2017	6

Figure S1. Reservoir elevations through time highlighting timing and duration of draining to streambed. A) Annual Fall Creek Reservoir elevations (dotted line) by day from 2006 (top) to 2017 (bottom), streambed reservoir level is shown as a solid red line (680', 207.3 mamsl). B) Photo of 2017 streambed draining. C) Duration of streambed exposure (elevation <207.6'). *Note: 2012 was considered 'at streambed' from Dec 9th through Dec 15th (6 days) though high inflows precluded the reservoir dropping below 207.6.

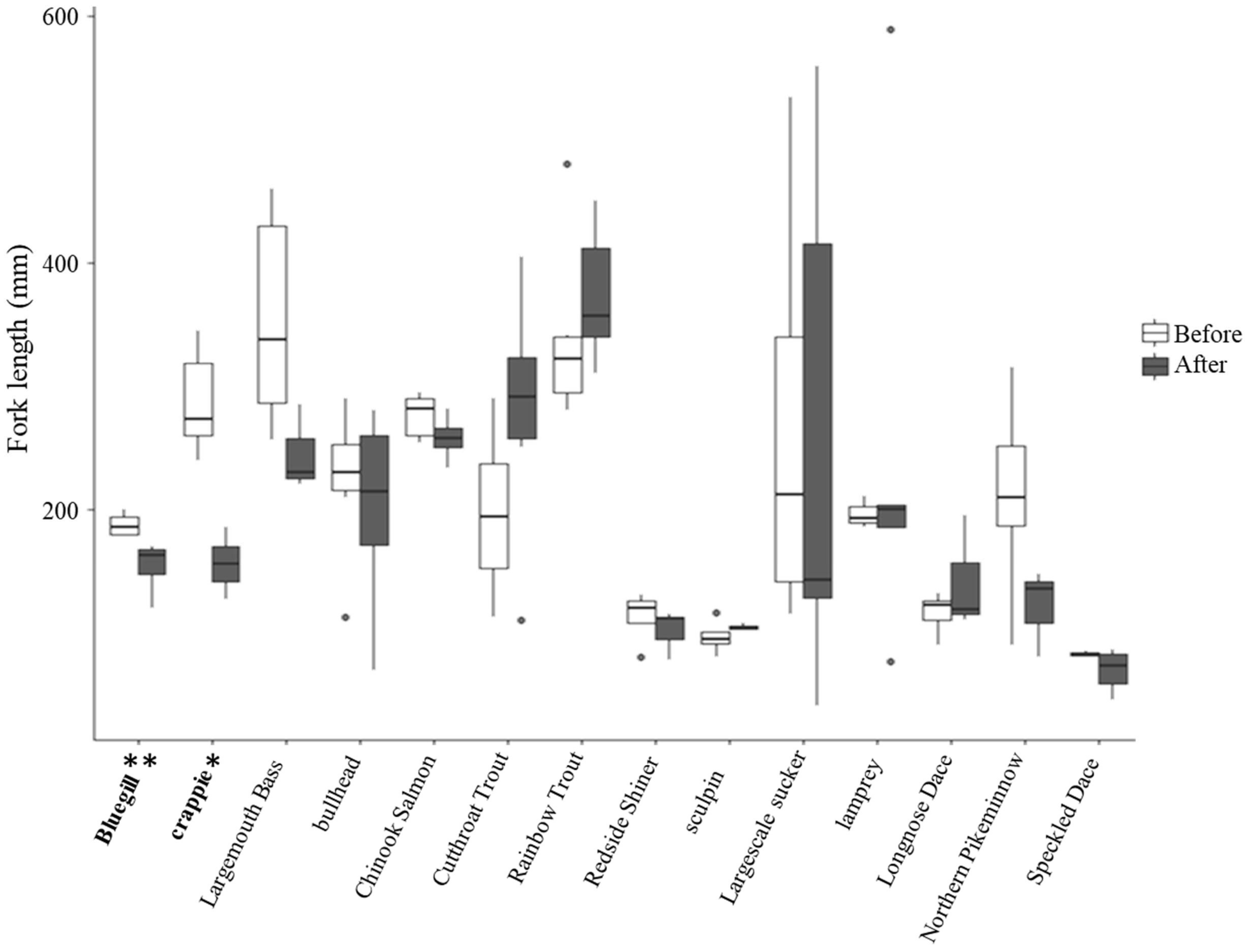

Figure S2. Annual maximum observed fish sizes by species before (open box; 2006-2011) and after (gray box; 2012-2017) initiation of streambed draining events in 2011. Bolded taxa with stars were significantly different after contemporary streambed draining. Unpaired Wilcoxon Test *R*, $P < 0.01$ (*) and $P < 0.001$ (**).

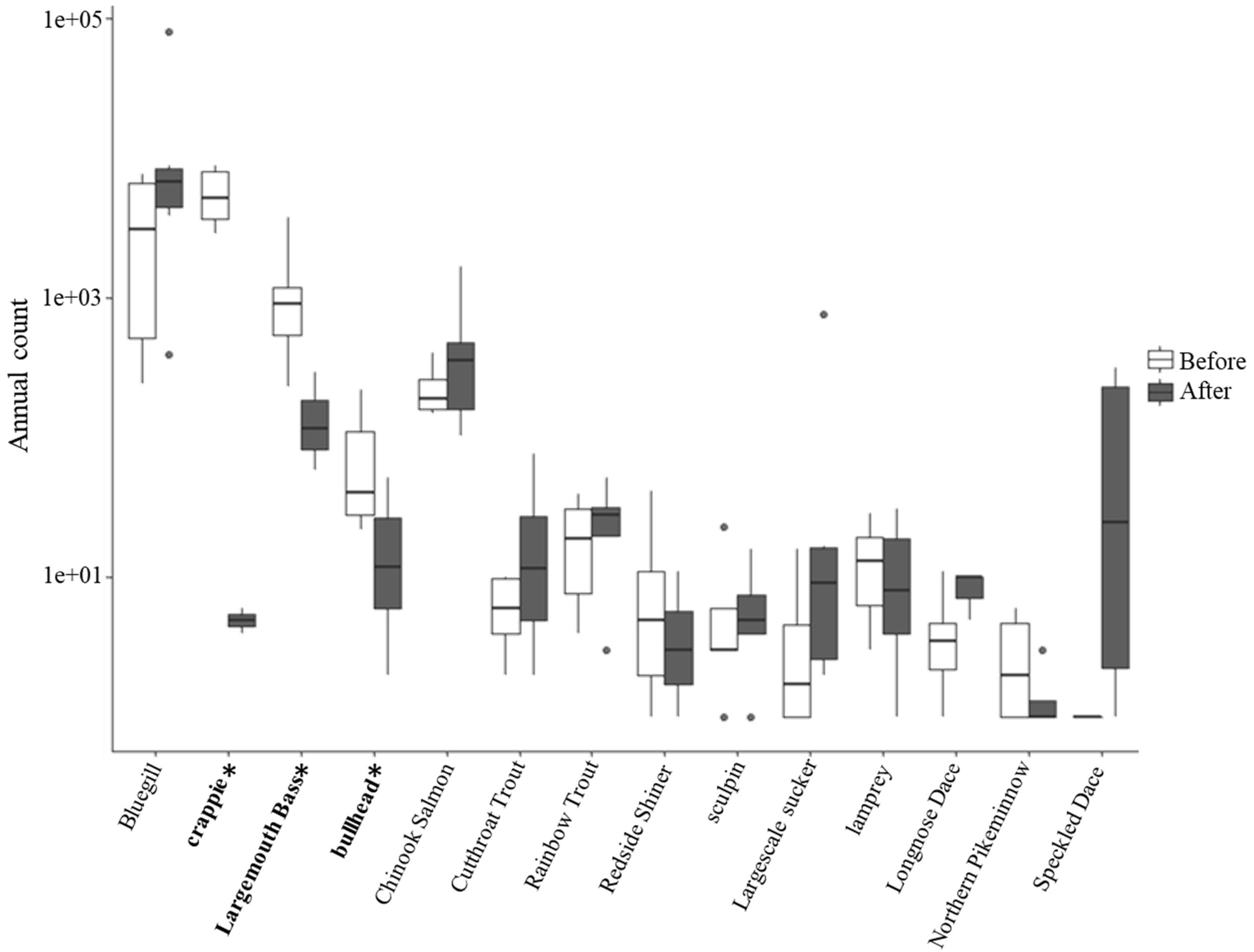

Figure S3. Annual count (log scale) of juvenile Chinook Salmon and other fishes before (open box; 2006-2011) and after (gray box; 2012-2017) initiation of streambed draining events. Bolded taxa with asterisks were statistically significantly different after contemporary streambed draining. Unpaired Wilcoxon Test *R*, $P < 0.01$.
